# Intra-peritoneal Adhesions

**Published:** 1885-07

**Authors:** B. E. Hadra


### INTRAPERITONEAL ADHESIONS.

Dr. Hadra, of San Antonio, Texas, has a paper in the *Journal of the American Medical Association*, June 20th, on "Intraperitoneal Adhesions in Relation to Tait's Operation," which is, in our judgment, one of great value. This paper was read before the section on gynecology, at the National Medical Convention, at New Orleans, in May last, and was discussed by Drs. Battey, Sutton, Marcy and other distinguished gynecologists, who attached much importance to the features which complicate the operation for removal of the ovaries, or ovaries and tubes, and which Dr. H. here calls attention to.

Dr. Hadra takes the ground that the majority of cases, which require Tait's or Battey's operation, require *something more,* and that "something more" is the breaking up of adhesions, wherever they exist, within the peritoneal cavity. The inflammatory process, which results in the condition for which the removal of the tubes, or ovaries, or both, is recommended or practiced, does not confine its ravages solely to those organs, but frequently glues together contiguous parts of the viscera, anywhere within the cavity. And, therefore, Dr. Hadra points out, it is necessary, after removing the tubes, or ovaries or ovaries and tubes, *if their removal be found necessary*, on making the exploratory incision, that the hand should be passed freely about in the cavity to search for, and when found, to break up said adhesions. But it not infrequently occurs that this breaking up of adhesions, with the hand, is the most important part of the proceeding; or, rather, it has been found, in cases where the removal of the tubes had been determined upon, and a laparotomy made accordingly, that the tubes were in good condition, and that what was needed was *only* the loosening of adhesions, which existed between adjacent parts of the serous membrane; and in those cases the tubes and ovaries were left in tact, the wound closed, and the patient made good recovery.

Dr. Hadra cites cases illustrating his position. We had the satisfaction of hearing this paper read, and discussed, and we were proud of Texas, when Battey and other great authorities on abdominal surgery, took such interest in its discussion, and acknowledged its value.

Dr. Hadra goes further and calls attention to intra-peritoneal adhesions *above* the pelvic organs, and says :

"A peritonitis, once set up, is liable to deposit its poison anywhere within the sac, and to cause circumscribed adhesive inflammation anywhere. This fact, which is well known to pathologists, has been somewhat neglected by gynecologists. A woman has an inflammation after dysmenorrhœa or in childbed, not severe enough to claim the dignity of peritonitis ; nevertheless, it is such, and, after a while, when she has been pronounced well, she will complain of pain in the abdomen, either all over, or only in a limited spot, most generally in the left epigastrium. These symptoms grow in intensity until we are induced to make Tait's operation, when we are surprised to find the uterine appendages normal. Sometimes massage, which we know can cure adhesive inflammation, will give relief."

* * * * "We can further understand that the peritoneal coverings of liver, spleen and stomach become involved, and in looking through Tait's list of laparotomies, we can readily see that the marvelous cures, of liver and spleen affections, are the result of *unavoidably breaking up adhesions* in the attempt to examine those organs."

"A case, the history of which I will give below, taught me that in some forms of latent peritonitis are strings, of lymphatic nature, which run between the different surfaces of the peritoneum, and which filaments, I am satisfied, shrink up after death, but during life *are the vehicles of lymph and other plastic and irritative material.* [Italics ours.—Ed.] These lymphatic strings might be the first stage of adhesive peritonitis, gluing together different structures and surfaces, or they might persist in this form, and give rise to many of the most common complications enumerated before."

"The history of the case, upon which I base my deductions, is as follows: A Bohemian woman, married eight years, has had two children, the last three years ago, since which time she has been an invalid; menstrual function has not been resumed since, constant pain over the entire abdomen, especially in left side. On examination, womb was found simply atrophied, nothing else. Laparotomy; about two tablespoonfuls of dark serum; ovaries normal, tubes seemingly healthy, (afterwards found closed, and mucous membrane thickened,) ovaries and tubes were removed, as Tait's operation was intended. Still, having my mind on the mentioned peritoneal adhesions, I introduced my hand under the omentum, and swept it over the whole anterior surface of the bowels, repeating this manœuvre three or four times, and each time my hand was covered with a *large number of transparent filaments resembling cobwebs.* The abdomen was closed, and the woman made an uninterrupted recovery."

Viewing the matter in this light, Dr. Hadra proposes, in the future, to make an incision, and then do what is found necessary for the relief of the woman, whether it be removal of ovaries, or ovaries and tubes, *together with* breaking up of adhesions, or whether it be breaking up of adhesion alone, if the ovaries and tubes are sound; and he intimates that there is too much of a *fashion* to remove those parts, for the eclat of having made a "Tait's operation." He suggests the following steps in the operation: "Laparotomy; minute examination of all the pelvic viscera, with special attention to adhesions; breaking them up; insinuating hand upwards with sweeping movements between omentum and bowels, and between omentum and parietal peritoneum. These movements should be made thoroughly, sweeping over all the surfaces, especially the side and spot where was most complaint. Thus a new operation, freeing the peritoneum throughout its entire area, will have been performed, and I hope, with the fullest benefit. Particularly should this operation be tried in young women, to save, if possible, the functions of generation." The Doctor closes by saying: "Let the operation conform to the conditions which are revealed by the laparotomy." Sound advice.

This paper is destined to exert a telling influence on the abdominal surgery of the future, and we look to hear of it in reports from "beyond the seas."

